# Local governance of the 2014 ebola Epidemic: a PhD synthesis

**DOI:** 10.1080/16549716.2024.2411742

**Published:** 2024-10-11

**Authors:** Sabine Iva Franklin

**Affiliations:** a Africana Studies Department, Wellesley College, Wellesley, MA, USA; b Westminster Development Policy Network, University of Westminster, London, UK

**Keywords:** Public health goods, demand-side barriers, traditional local institutions, polycentric governance, community-led action

## Abstract

**Background:**

The doctoral dissertation examines how local response efforts were integrated into overall emergency management.

**Objectives:**

It seeks to understand the role and effectiveness of community-based actors in addressing collective action problems

**Methods:**

Sixty-seven semi-structured interviews were conducted from January to July 2017 in Liberia and Sierra Leone. Key informants include healthcare workers, traditional leaders, and community stakeholders, such as non-governmental organization representatives and volunteers.

**Results:**

Findings show that traditional and community leaders responded to the public health emergency via rulemaking, quarantine, travel limitation, healthcare referrals, health sensitization, and door-to-door contact tracing. These actions by local leaders helped to change behaviors and improve cooperation. Sierra Leone had 32.3% more Ebola cases than Liberia but 18% fewer deaths. Sierra Leone had integrated traditional and community leaders before the scale up of international aid resources.

**Conclusion:**

This suggests that actions taken by traditional and community leaders improved overall efforts, and in some areas, before scaled-up humanitarian interventions. Bilateral engagement with local community actors should be integrated in every public health response to improve cooperation, and it should be done before an intervention is conceived and executed.

## Paper context

- **Main findings:** Bottom-up legislation and community-led action were significant in containing the EVD spread in Liberia and Sierra Leone.
- **Contribution to knowledge:** Theoretical contribution centers on the governance patterns of Traditional Local Institutions. Evidence-based contribution was the observation of polycentric governance patterns of demand and supply-side barriers between traditional, state, and aid institutions.
- **Global health impact for policy and action:** Policymakers should contextualize soft factors such as trust, which can hamper technical advice. Any intervention should include bilateral engagement with local community leaders.

## Background

This paper is a synthesis of a doctoral dissertation successfully defended in November 2019 at the University of Westminster in London, UK [1] The research is a comparative analysis of local response efforts to the 2014 to 2016 Ebola Virus Disease (EVD) epidemic in Liberia and Sierra Leone. Although it has been a few years since its publication, the recent COVID-19 pandemic demonstrates that there is a need to improve public health emergency management in local communities. Many nations, rich and poor, struggled to gain widespread cooperation with emergency management rules, such as masking, social distancing, travel restrictions and vaccinations [2,3]. This lack of cooperation is what economists and political scientists call: *collective action problems* and can perpetuate disease spread during an outbreak.

Understanding why collective action problems arise during a public health emergency and how to address them can reduce disease spread and, hopefully, deaths. The traditional approach focused on the rapid deployment of biomedical resources to ‘treat or cure’ our way out of an outbreak. This is the supply-side of a public health intervention, and it is assumed that people will always want the biomedical or clinical resources and information provided. Or that individuals will adopt biobehavioral changes, such as social distancing, to prevent disease transmission. These are the assumptions made of the demand-side of an intervention. However, during the EVD outbreak, considerable resistance to state restrictions and health resources arose, with many experts and pundits citing lack of education or understanding of science and Western medicine as the reason [1]. These were simplistic and stereotypical assumptions that did not examine the complex dynamics of the demand-side barriers. Factors such as trust, institutions, and social engagement helped to resolve collective action problems.

This dissertation offers insight into what happened, why collective action problems arose, and how community-based institutions addressed these barriers. This research is part of a portfolio of projects undertaken as a postdoctoral fellow at Yale University and Wellesley College to examine local community engagement during public health emergencies. This paper summarizes chapters 2 through 5 of the dissertation, and the section headings correspond with each chapter headings in the dissertation.

## Literature review

Chapter two of the dissertation is the literature review and the theoretical framework that is put forth to address the gap in the literature. It is divided into 8 sections, including the introduction and conclusion. The first section discusses the concept of institutions with a normative definition of rules and norms that shape our behaviors. These can be formal rules, such as criminal law or informal rules, such as how to behave during a job interview. There are over 3 decades of research across the social sciences that examine institutions and their impact on society. Economists, such as Douglass North, argue that state institutions that adopted capitalist economic policies are responsible for modern and developed societies in the West. He argues that these state institutions have high institutional quality that brought economic growth. His research was adopted for neoliberal good governance reforms in developing countries that encouraged austerity and privatization of public services, such as healthcare [1].

Sections 2.3 and 2.4 challenge this argument because it marginalizes non-state actors and institutions, such as traditional leaders and community rules and norms that are prevalent across sub-Saharan Africa. The dissertation coins the term, ‘Traditional Local Institutions’ (TLIs) to describe them. TLIs were designed outside pro-market rules and are based on pre-colonial norms and culture, such as restorative justice and social cohesion. This section also reviews multidisciplinary literature written by social scientists, such as Paul Richards and Daron Acemoglu, who are critical of TLIs in West Africa, with the former advocating for these institutions to be abolished. The dissertation stands against these critiques. Thus, section 2.5 argues that ‘good governance’ should not only be measured in outcomes for the economy but also in outcomes for social welfare. This sets the stage for examining the role of TLIs in the West African EVD epidemic. However, first, we need to understand how disease outbreaks in low-income countries have previously been addressed by state and international NGO institutions.

Section 2.6 unfurls the problem of how public health emergencies are normally responded through a biomedical and clinical lens in a top-down approach from state institutions. These responses usually focus on supply-side management of the interventions such as bringing medical equipment, volunteers, and clinical information to ‘treat’ our way out of a disease outbreak. This section specifically discusses the World Health Organization (WHO) communication (COMBI) toolkit that emphasizes risk communication to change behaviors for ‘desired outcomes’ and gives examples of its use, such as the 2007 Marburg virus outbreak in Angola and the 2000 EVD outbreak in Uganda [1]. This public narrative says that Western agencies and state governments coordinated a response via a biomedical paradigm for humanitarian assistance. However, resistance or ‘undesirable’ behaviors from the community, such as at-home caregiving or funerals, were obstacles to the ‘solutions’ being provided by Western agencies. Or, in other words, local community norms had poor institutional quality and needed to be replaced with Western-derived norms and resources to successfully resolve these outbreaks.

The final sub-section engages with multidisciplinary literature of the 2014 EVD epidemic and the perspectives of the social mobilization strategies employed. Fairhead (2016); Leach (2015); Nunes (2015); Pailey (2017); Wilkinson et al. (2017); Wilkinson and Leach (2014) examine how structural violence from colonial legacies degraded public health and healthcare administrations in West Africa. Thus, the ‘risky or undesirable’ behaviors such as burials and caregiving continued because state and aid institutions have and continue to fail local communities [1].

The second group of literature collectively discusses supply-side intervention coordinated by the WHO and international NGOs. It claims to engage in dynamic communication strategies, training of local volunteers, and a correction of information asymmetry (i.e. rumors and misinformation) to resolve collective action challenges in Liberia and Sierra Leone.

However, this is a biomedical reductionist approach of pathology and epidemiology and does not center the people who are affected by an epidemic (demand-side). In a WHO report regarding Liberia, ‘Intensification of technical interventions, like increased laboratory capacity, more treatment beds, and a larger number of contact tracing and burial teams, will not bend the curve in the absence of community engagement and ownership’ [1]. In other words, simply increasing the supply-side of public health goods will not end a disease outbreak, there is a demand-side where community members need to be integrated in response efforts.

As chapters 4 and 5 will discuss, much of the information asymmetry was caused by state institutions giving incorrect information and procedures, lack of information in some areas, and being slow to update information. This eroded confidence and many people avoided the healthcare centers and EVD treatment centers. In other words, poor governance (or institutional quality) created demand-side barriers. The reason for the lowered demand is not because people do not care about their health, but rather continued poor-quality of care, nosocomial transmission of disease, and poor leadership led to fear and mistrust.

However, these individual behaviors lead to costs and externalities. In economics, ‘cost’ is inherently negative because it assumes a loss, such as a loss of good health (illness) or a loss of life (EVD death). ‘Externality’ is a neutral term referring to a third-party receiving a cost or benefit indirectly. Using an example from Becker and Becker (1997) if I as a non-smoker spend most of my days in an enclosed space with a smoker, this could lead to a private cost for the smoker (this person gets sick), a social cost (higher insurance premiums because of tobacco-related illnesses), and negative externalities (I get sick too). By mobilizing to resolve demand-side barriers, this can lead to positive externalities such as reduced illnesses or costs [1].

The third group of literature analyzes the hidden narrative of the involvement of traditional and community leaders. This comprises Abramowitz et al. (2015); Bedford and Miller (2017); Bonwitt et al. (2018); Goguen and Bolton (2017); Parker et al. (2019); Perry and Sayndee (2017); Richards (2016); Van der Windt and Voors (2020) [1] .Papers on Sierra Leone discuss the role of paramount chiefs implementing *bylaws* as emergency management regulation and, in Liberia, how some non-state actors were able to organize a response in their local communities. Much of this work focuses on the positive attributes of these actors to reduce social costs and correct information asymmetry. However, there are some scholars who oppose this hidden narrative that traditional and community leaders made a positive contribution. Boland and McKay (2018); Enria (2017); Wilkinson et al.. (2017); Wilkinson and Fairhead (2017) argue against the authoritarian nature of the *bylaws* in Sierra Leone and are skeptical whether these helped resolve collective action challenges [1].

Indeed, there is no way to assert that every paramount chief or community leader was 100% effective in their role. However, no state- nor NGO-led intervention can be described as 100% effective either. What is known is that the first case of EVD occurred in December 2013 and it was not confirmed and announced by state institutions in Guinea until March 2014, then Liberia and Sierra Leone in April and May 2014, respectively. The WHO declared a global health emergency in August 2014, which scaled up aid resources. Thus, there is a large gap in the timeline, and it would be absurd to claim that Western-led intervention in the Fall of 2014 was the first meaningful act to stop EVD.

## Timeline

In order to reconstruct the narrative, the timeline is divided into two periods: phase one from December 2013 to September 2014 and phase two from October 2014 until EVD was declared over in June 2016. The WHO reports phase one starting after their declaration of a global health emergency. However, this assumes nothing meaningful happened before August 2014. The proposed timeline is designed from the informants’ view of notable events during the first several months before the international community mobilized aid resources. By creating this timeline, it decolonizes the narrative of the outbreak by centering the key events from the perspective of local actors. This is inspired by Chakrabarty’s work on decoloniality and global history [1]. Most of the social sciences, public health, and gray literature give a top-down perspective starting from late 2014 with some notable exceptions mentioned above.

## Methods

This section uses the ENTREQ toolkit to synthesize the methodology from Chapter 3 of the dissertation [4]. The research design is from a toolkit for qualitative research created by the Critical Appraisal Skills Programme for public health research [5].

The literature search was an iterative and multidisciplinary process that included academic and gray literature across African studies, economics, law, medicine, political science, and public health. Inclusion of sources was very broad and the literature that was excluded ended up being academic literature that did not fit the final theoretical scope of institutional quality, social costs, and polycentric governance. From 2016 to 2019, I conducted literature searches through google scholar, PubMed, google news, JSTOR, and recommendations from supervisors and colleagues. I often used keywords such as ‘Ebola virus disease’ ‘community engagement’ ‘social mobilization’ ‘Institutional Quality in Traditional Institutions’ ‘Institutional Quality and social costs’ ‘Polycentric governance in health’ ‘information failure’ ‘Community participation’, I have also researched specific authors that have published along similar themes of community-based institutions, such as Paul Richards, Katharine Baldwin, and David Harris. I searched for reports and media from NGOs that responded in West Africa, such as Samaritan’s Purse, Red Cross, ICRC, Save The Children, MSF, and the WHO. I also searched the archives of local newspapers in Guinea, Liberia, and Sierra Leone. Throughout the course of the project over 1000 bibliographic entries were recorded in Zotero, a library software, and about 500 of these entries were used in the bibliography of the dissertation. Apart from my supervisors’ recommendations, I determined which sources were used in the final manuscript based on content, theoretical relevance, and clear methods whether experimental, qualitative, quantitative, or mixed.

Data collection was conducted in Liberia and Sierra Leone from January to July 2017. Sixty-seven one-on-one semi-structured interviews were conducted with healthcare professionals, community stakeholders, such as NGO representatives, traditional leaders, and government workers. Interviews were transcribed and entered in the Nvivo software for coding and analysis. I primarily led the coding; however, after consultation with my supervisors, I would return to do more coding. I used the search function on the Nvivo software to broadly match text for coding. Each country had its own separate folder in Nvivo and the process of coding was repeated for each folder, using the same keywords. Three themes were pulled from the coding: ‘Government’s Response and Community Reaction,’ ‘Local Institutional Intervention,’ and ‘Governing the Outbreak’. However, the codes that make up the themes are different for each country. For example, in Sierra Leone, the second theme mainly consisted of the code: *bylaws*. In the Liberian dataset, it consisted of the code: *communities organizing*. The research process was a mix of deductive and inductive methods, as I started the process wanting to understand local governance, but after data collection and analysis, I had to read new material to refine my understanding of governance and institutions to interpret the dataset. The next section synthesizes the findings from chapter four of primary data collected from interviews and secondary epidemiology data.

## Findings

### Baseline

Key informants were asked about the healthcare system after the civil wars ended in the early 2000s, but before the EVD epidemic. There was a universal description of very poor healthcare service delivery and scant infrastructure of any kind in both countries. Healthcare workers and some community stakeholders were aware of the low levels of trust in government services before EVD, which hampered emergency management. These are demonstrated in the quotes below. The remaining sections are organized thematically.

I think there [has] always been a distrust of government, so that [was] also one of the weaknesses before the Ebola and one of the weaknesses during the Ebola crisis. There’s been a distrust of government. If the government says, ‘There is Ebola,’ nobody believes the government. It’s trust. So, government can come and say, ‘We’re doing this,’ and everybody will say, ‘That’s not true,’ there’s a mistrust, OK. So, communities do not trust government. Zero. Anything the government comes up with, they don’t believe it, because they know it never, never happens in Liberia. So, because of that, communities are more likely to align with international organizations to be able to accomplish. (Community Stakeholder, Montserrado County, Liberia) [1].

From their training, college, and their nursing schools, they have not been receiving enough training, so that when they come to the field, people trust them and know what they are doing. I think for quite a while now, it has been losing credibility because people have been accessing the health facilities without being cared for, without having the correct service that they want. So, their needs have not been met. So, there is a lack of trust in the health sector. That was quite evident during the outbreak. People did not go to the hospital because they think they may get the infection in the hospital. (Healthcare Worker, Bo District, Sierra Leone) [1].

### Theme 1: government response and community reaction

This theme explores the informants’ perception of how state institutions responded to EVD during phase one. Given the baseline of attitudes towards the central governments, there was an expectation that centralized policies would fail. Healthcare workers habitually have poor working conditions, where clinics and laboratories are poorly supplied, as explained below:

Like, when they supply us, we just have to manage because when it is finished, we will catch a hard time to get it. So, when it is here, when it is available, we have to take our time [in] how we will use it. We don’t really use it, like, take for example, if you get about, maybe, they just brought about fifty of these gloves. OK [but] now for NCH we can use more gloves. You want to touch patients, you want to do this, you want to do that every now and then [and] we have to change gloves. So, we have to take our time [in] how we really use it, so we can’t finish it. Because when it finishes, we have difficult time to get another supply. (Healthcare Worker, Montserrado County, Liberia) [1].

The supply chain was highly centralized, meaning it could take several weeks or months to receive basic personal protective equipment (PPE), such as masks. Thus, healthcare workers used supplies judiciously, if it is not believed that a patient has an infectious disease or needs a procedure that is not too bloody, then gloves are likely not used. Consequently, patients with vague flu-like symptoms were not seen as threats. This state institutional failure resulted from poor dissemination of information, specifically, the case definition for EVD. For example, most communiqués described patients who would have a history of eating *bushmeat*, which means wild game. However, the type of wild game present in West Africa is not known for carrying or transmitting EVD. Human-to-human transmission was not emphasized in official communications even though this was the primary epidemiology chain in West Africa [1]. So, ill patients who did not eat *bushmeat* in the last few days were not suspected of having EVD. A second example of information asymmetry was the more graphic sign of bodily hemorrhaging. Hemorrhaging is a rare sign that occurs in less than 5% of EVD patients and it typically occurs when someone is at the end-stage of illness [6]. The majority of EVD cases had vague flu-like symptoms that were often misdiagnosed as Malaria or Cholera. These factors led healthcare workers to treat patients without gloves or isolating infectious cases.

However, the spike in deaths from ‘Malaria or Cholera’ raised suspicion, panic, and mistrust. Individuals who did not eat wild game became ill and some died, while some who did eat wild game did not become ill. These observations within communities led to perceptions ranging from incompetency of healthcare workers to conspiracies of nefarious activities.

### Theme 2: local institutional intervention

The second theme describes how community-based actors mobilized in response to EVD. This was largely due to the perception of a poor government response and local communities needing to act as a result. This is described in the below excerpt:

The community took the initiative to organize themselves, like for us, we organized ourselves. We put ourselves in a group, and we go from house to house; we find sick people, and we make sure those sick people were transferred to the hospital for proper care. So, as for me, my community, the [redacted] community we didn’t experience any outbreak, and there was no symptom of Ebola in the [redacted] community. Because we mobilized ourselves into a group without anybody helping us. (Community Leader, Montserrado County, Liberia) [1].

Liberian key informants discussed a variety of strategies that local communities employed. Above, a community member explains how he organized volunteers to go door-to-door to provide information about EVD, distribute hygiene supplies such as soap, and hospital referrals. Referrals were practiced as a volunteer accompanying a sick person to the local clinic for moral support. Some areas also created checkpoints to prevent nonresidents from entering and guests who were visiting were asked to return to their homes. In areas that self-mobilized, key informants reported positive impacts such as reduced cases, or in this specific experience of this informant from the Monrovian suburbs: no EVD cases.

The first district to report EVD in Sierra Leone was the Kailahun district, which is in the easternmost part of the country and shares a border with the northern Liberian county of Lofa (where Liberia’s first EVD case was identified) and Guinea’s southeastern prefecture of Guéckédou, where the first EVD case of the West African epidemic was identified.

The paramount chiefs from all chiefdoms in Kailahun met to discuss the effects of the outbreak, government response, and how to intervene. This resulted in a series of emergency management regulations that chiefs have the authority to pass, monitor, and enforce called *bylaws*. *Bylaws* consisted of quarantine/isolation, banned at-home caregiving, mandated healthcare referrals, banned funerals, initiation rituals, and mass gatherings. Violators would face a fine of 500,000 Le, which was roughly one month’s salary during this time, as this traditional leader explains below:

They told us, ‘Whoever has a sick person at home should report to the nearest PHU or main practitioner that is closest to you.’ If you don’t do that, and they realize that, they will fine you 500,000 (Le). And Mende people will fear fines so much. They told us that we should not touch corpses. When there is a corpse, a dead person, we should call [a] medical practitioner. You know the infection between your loved ones - when your loved one is dead. You want to touch or clean it or so, but when your loved one is dead, we are infected by that, so we never touch any dead one. And travel was canceled, because if you traveled, they wouldn’t allow you to sleep in any other places. So, I think that saved us greatly … So the 14 paramount chiefs in Kailahun district came together to put the bylaws together and communicate that to the government, so that was in effect. So, all the districts in the country started implementing it, which worked effectively. (Traditional Leader, Kailahun District, Sierra Leone) [1].

Stakeholders in the neighboring district of Kenema heard about the implementation of the *bylaws* and community stakeholders (including the chiefs) met to discuss implementing similar emergency regulations, as this NGO representative below explains.

No, it was from the bottom to top. We had agreed on these things in our meetings, and we decided to operate it. There was a time when we met with someone from Kailahun, [redacted] because they were the first people to start making bylaws. We called them to come and share their experiences with us and that one was very good. So, we had to present ours, and he presented theirs and told us how they were able to overcome a lot of difficulties with the bylaws … . (Community Stakeholder, Kenema District, Sierra Leone) [1].

There is a consensus among key informants from the epicenter of the Sierra Leonean outbreak that demand-side barriers reduced after TLIs intervened with emergency *bylaws*.

### Theme 3: governing the outbreak

This final theme uses a polycentric perspective to understand how community-based institutions govern demand-side and aid institutions govern the supply-side, with state institutions fielding both.

Liberian informants discuss how fear and stigma during phase one led many to stop working, and as a result, many healthcare centers closed. This hampered access to care for EVD and non-EVD cases, resulting in at-home caregiving, no treatment, or traveling greater distances to find an open center. All of which leads to greater negative externalities that perpetuate EVD spread. Many healthcare workers believe the scale-up of aid resources in phase two, helped to re-open centers. The supply-side of public health goods not only brought clinical resources but also training (i.e. correcting information asymmetry) and enhanced wages.

Yes, they brought instruments and a lot of things that will help us to protect ourselves from getting Ebola. And we got a lot of training from the Ebola outbreak and right after the Ebola. During and after the Ebola, we had a lot of training concerning Ebola. How to care for patients, how to triage patients at the gate before they come in, to know between the patients who come first and who last, who should be first to be treated. At least the Ebola helped us, we went through a lot of workshops, and it was successful. (Healthcare Worker, Montserrado County, Liberia) [1].

However, for community stakeholders, they did not share the same experiences with aid institutions.

Even during the Ebola, what we noticed was that there was some money allocated for the communities, but it was processed through representatives from the various districts. Like for this district, where [redacted] is the representative, we learned the money was passed through those people for the district to fight the Ebola, but we didn’t see that in our own place. The only thing we noticed was that when we first launched our community Ebola team, invited stakeholders, we invited [redacted] who is the representative for this district, and he came. When he came, he first started with L$ 10000 and then we invited Robert Sirleaf [son of President Johnson-Sirleaf] and he came in too and gave us [a] few buckets and some rice, to say you have a special link with the government and the community, [but] it didn’t happen that way. Then, from there, what we did on our own was, we bought buckets and came to give them to people too, to wash their hands. We got in contact with a lot of NGOs, but they couldn’t come to our aid, but people benefited too, other people benefited because they have the Ebola there. So, they have NGOs helping that community apparently, but they didn’t come to us because we didn’t have [an] Ebola outbreak [here] apparently. Maybe this is the reason assistance was not coming to us. (Community Leader, Montserrado County, Liberia) [1].

The president of Sierra Leone declared a state of emergency in August 2014 and asked all paramount chiefs in the country to implement emergency *bylaws* as the chiefs did in the Kailahun and Kenema districts. This made emergency management more efficient and the authority of TLIs gave protection to healthcare workers to enter communities safely, as described by this healthcare worker below:

Bylaws from area to area…you know when announcements go through parliament it is a process and a longer time, but with bylaws it is quicker here with the local chiefs. I think that is why the government also dealt with the local chiefs later, that they should go and man checkpoints in their localities during the Ebola. Once they were involved, they created bylaws and created a level playing ground for the medics to come and work protectively. (Healthcare Worker, Kenema District, Sierra Leone) [1].

The experiences of TLIs, healthcare workers, and aid institutions are mixed. Some key informants reported to have flattened the curve before the involvement of aid and state institutions, while other informants reported some collaboration with state and aid institutions in phase two to help maintain a flat line. This could be indicative of aid resources concentrating in the capital area of Freetown and not penetrating the rural provinces.

They created the NERC, the National Emergency Office was created, and at the district level, the District Emergency Operations office was created; where there were other people that were recruited, and they were managing the Ebola. Like for this district, there was some conflict among the health workers, because at the time the emergency unit was created, we had already done away with the Ebola here. It was not actually here again. Then, you take the responsibility away from the health workers that they were not up to expectations. So, most of us were annoyed because we had already done the fight. Instead of compensating us or give us thanks, you [come] in the manner that you bring people to come and take over for us. (Healthcare Worker, Kailahun District, Sierra Leone) [1].

### Secondary data: WHO statistics

EVD cases and fatality rates collected by the WHO were triangulated for this research. Sierra Leone had 14,124 EVD cases and 3,956 EVD deaths, whilst Liberia had 10,675 EVD cases and 4,809 EVD deaths throughout the epidemic [1]. This presents a case fatality rate of 28% in Sierra Leone and 45% in Liberia. Thus, the epidemiological data suggests a significant divergence that could be explained by environmental factors. The next section synthesizes chapter five of the dissertation, which contextualizes these findings.

## Discussion

Chapter 5 discusses how the interventions and strategies executed in Liberia and Sierra Leone during phase one (i.e. before the WHO’S declaration) challenges the dominant narrative of Western heroes solving an ‘African problem’ that morphed into a global threat [1]. This discourse presents a stereotypical narrative that does not strongly challenge the competency and leadership of state and aid institutions. Moreover, the data also challenges the mainstream narrative of EVD denialism and lack of cooperation with emergency mandates due to religion, superstition, or illiteracy. Rather, many key informants believe that state institutions are apathetic to the suffering of marginalized communities, especially when regional politics come into play. The rising number of deaths, closed healthcare centers, and panic led community members to act and implement local emergency management procedures.

Comparing the data, Sierra Leone had a more organized and near universal approach through the *bylaws* implemented and monitored by TLIs, whereas community organizing observed in Liberia varied greatly. This can explain the significant difference in the 45% case fatality rate in Liberia and 28% rate in Sierra Leone. Moreover, Sierra Leoneans identified 32.3% more EVD cases than Liberian counterparts. This suggests that the integration of TLIs into emergency management also improved contact tracing and referral into care for early treatment, which increased survival rates.

An unexpected finding was how these policies incentivized workers to leave the healthcare centers. Given the lack of supplies, such as PPE and equipment, it is rational that healthcare workers stopped providing care, especially after seeing many of their colleagues die. Furthermore, wages for healthcare workers in both countries are not reliable. Sometimes they receive their ‘monthly’ salary every few months, and that may or may not include the entire back wages owed to them. In Sierra Leone, many healthcare workers are skilled ‘volunteers’ meaning they hope to one day be officially hired and placed on payroll, which could take years. Thus, the term ‘volunteer’ is a misnomer as this is a form of labor exploitation.

In the Western world, the decision to stop working would arguably be a private cost, because of lost wages. However, in the West African epidemic, clinics were sources of disease transmission and workers were not protected nor fairly compensated. Thus, considering this context, withholding labor was a private benefit. Consequently, this led to massive negative externalities as local communities lost access to care [1]. Research that is currently being conducted in Sierra Leone further examines this problem of labor and wages in the healthcare sector, especially within the context of the COVID-19 pandemic.

The concept of demand-side barriers is complex, and not all demand-side barriers could be solved by community-based institutions. For example, the height of the EVD epidemic was during the rainy season, when unpaved roads turn into muddy rivers. This severely hampered aid resources from penetrating rural healthcare facilities. Furthermore, there is a lack of infrastructure for infection prevention control such as running water at healthcare facilities; these are widespread problems that should be addressed by state institutions.

## Conclusions

It is not if, but when, a disease outbreak will occur; and there will always be challenges with supply-side management and interventions. This research examines the benefits and limitations of integrating community-led action into emergency management. Moreover, while the context of the study are low-income countries with fragile institutions and systems, recent experiences with the COVID-19 pandemic demonstrate that collective action problems can happen in any nation [2,3]. Thus, there is urgency for more research that identify and address demand-side barriers that can impede public health interventions.
